# Effect of rituximab dose on induction therapy in ABO-incompatible living kidney transplantation: A network meta-analysis

**DOI:** 10.1097/MD.0000000000024853

**Published:** 2021-03-12

**Authors:** Hee Ryong Lee, Kipyo Kim, Seoung Woo Lee, Joon Ho Song, Jin Ho Lee, Seun Deuk Hwang

**Affiliations:** a Division of Nephrology, Department of Internal Medicine, Leesin Hemodialysis and Intervention Clinic, Busan; b Division of Nephrology and Hypertension, Department of Internal Medicine, Inha University School of Medicine, Incheon, Republic of Korea.

**Keywords:** ABO-incompatible kidney transplantation, all-cause mortality, dose of rituximab induction, graft failure, infection, kidney transplantation, network meta-analysis

## Abstract

**Background:**

Rituximab is an induction immunosuppressant essential for ABO-incompatible kidney transplantation (ABOi KT). However, studies on its dosing, which differs among countries and transplant centers, are lacking. Therefore, we retrospectively investigated the effectiveness of the induction dose of rituximab against patient mortality, graft failure, and adverse events.

**Methods:**

We included the studies referring to at least 2 of eligible induction doses (200 mg, 200–500 mg, or 500 mg) of rituximab during ABOi KT and relevant outcomes such as patient survival, graft failure, and bacterial and viral infections. We performed direct and indirect network meta-analyses using Bayesian models and ranked different rituximab doses using generation mixed treatment comparison. Publications were retrieved using CENTRAL, MEDLINE, EMBASE, and Science Citation Index Expanded databases from 1970 to February 2020 and analyzed. The GRADE of network meta-analysis approach specified 4 levels of certainty for a given result: high, moderate, low, and very low.

**Results:**

Among the 4256 patients from 21 trials, glomerular filtration rate, graft loss, antibody-mediated rejection, T-cell mediated rejection, fungal infection, bacterial infection, and CMV infection did not differ among ABOi groups treated with different rituximab doses. The effect on mortality was significantly higher in rituximab 200 to 500 mg, and rituximab 500 mg groups (odds ratios [OR] 3.5, 95% CrI: 1.3–9.8, and OR 3.0, 95% CrI 1.1–9.8), but not in rituximab 20 mg group (OR 0.45, 95% CrI 0.036–2.5). The incidence of BK virus was significantly lower in the rituximab 200-mg group than in the other groups.

**Discussion:**

In ABO-incompatible kidney transplantation, low-dose rituximab is more efficacious than higher doses and reduces serious infection risks. Additional randomized controlled trials might be needed to confirm these findings due to small sample size.

## 1 Introduction

For decades, the outcome of ABO-incompatible kidney transplantation (ABOi KT) has not been significantly different from that of living donor ABO compatible KT (ABOc KT).^[1]^ Although protocols for various immunosuppressive agents have been developed, preoperative, or postoperative plasmapheresis (performed in the early stages of ABOi KT)^[2]^ may cause bleeding or surgical complications. Consequently, plasmapheresis is not performed after surgery, and there has been a reduction in the overall number of procedures.

Furthermore, rituximab (Roche Pharma AG, Reinach, Switzerland) is administered before transplantation to prevent the production of new antibodies, and maintenance immunosuppressants (e.g., tacrolimus, mycophenolate mofetil, and steroids) are commonly administered after induction immunosuppressant treatment immediately before transplantation.^[3]^ Rituximab is a chimeric monoclonal antibody against cluster of differentiation 20, a transmembrane polypeptide ligand on the surface of human B lymphocytes, to which rituximab attaches to exert its effect. In particular, apoptosis of immature and mature B cells is induced through antibody-dependent cellular and complement-dependent cytotoxicity. In addition, rituximab binds to Fc receptors and reacts to macrophage and natural killer cells.^[3]^ Rituximab exhibits its effects immediately after administration, and the effects last from 6 months to up to 2 years.^[4]^

Rituximab was first approved for the treatment of hematological malignancies and was initially used for indolent B-cell non-Hodgkin lymphoma and later for autoimmune disorders.^[5]^ Until 2004, splenectomy was used for B-cell depletion. However, because of the complications and side effects associated with the single-dose, administration of rituximab 5 to 7 days before transplantation has become the most common treatment method.^[6,7]^ Following the standardization of rituximab administration, dose reduction became the next concern in transplantation immunology. High doses of rituximab have been linked to various infections. In particular, the risk increases in patients with hematological malignancy, which requires repeated rituximab administration. However, data on the use of rituximab in rheumatological disorders or solid organ transplantation are lacking. Usual infections are bacterial, such as pneumonia and urinary tract infection^[8,9]^; viral, including cytomegalovirus (CMV), varicella zoster, herpes simplex, and hepatitis B^[10–12]^; and fungal.^[13]^

Rituximab is administered at various doses. However, recently, more centers have been using low doses^[7]^ because high doses increase the risk of infection. Moreover, efforts are being made to determine an optimal dose that is both effective and free of complications.^[14]^ Currently, studies on the effect of B-cell depletion and ABOi KT outcomes based on doses of rituximab are insufficient. Therefore, in this study, the effects of 3 doses of rituximab ranging from 200 mg to 500 mg were compared on graft and patient outcomes and the frequency of side effects. The doses prescribed for rituximab differ among medical centers, and although some report that high doses are more effective, other centers claim that similar effects are observed at low doses without risks of infection. Therefore, this study aimed to determine the appropriate dose of rituximab at each center so as to lead to more comparable outcomes between ABOi KT and ABOc KT procedures.

## 2 Methods

### 2.1 Ethics statement

All procedures were conducted in accordance with guidelines of the Preferred Reporting Items for Systematic Reviews and Network Meta-Analyses (PRISMA) statement (S1 Checklist). All analyses were based on previously published studies, and therefore, ethics approval and patient consent were not required.

### 2.2 Data sources, searches, and inclusion and exclusion criteria

**Figure 1:**
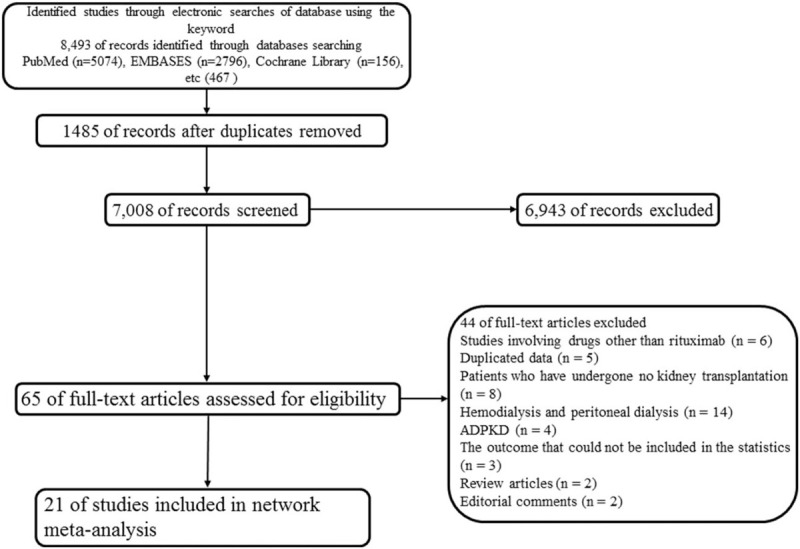
Flow diagram of systematic review (Preferred Reporting Items for Systematic Reviews and Meta-Analysis [PRISMA] Flow Diagram).

We comprehensively searched the following databases from their inception up to March 21, 2019: MEDLINE (using PubMed), EMBASE, CINAHL, Web of Science, and the Cochrane Central Register of Controlled Trials (CENTRAL) in the Cochrane Library. We searched for important keywords according to patient group and intervention (Supplement 1). The studies included in the review were on adult patients >18 years of age. Reviews, observational studies, and clinical trials that did not clearly define outcomes or did not report graft failure as an outcome were excluded. The search was limited to human studies but was not restricted to any particular language or publication date. Reference lists from all available boardboar articles were searched manually (Fig. 1).

### 2.3 Study selection and data extraction

The abstracts and full texts were independently evaluated by 2 researchers (SDH and JHL), and 2 reviewers extracted and re-evaluated the retrieved data. Any disagreements were resolved through discussions and consultations with another researcher (KK). The studies were divided into ABO compatible, placebo, and ABOi groups.

ABO compatible group refers to a group that used induction therapy to suppress T cells, a general immunosuppressant, because ABO type was matched between donor and recipient as the control group in this study. For comparison with the ABO compatible group, data from ABO mismatch patients having different ABO types between donor and recipient were required. Therefore, it was required that B cells should be suppressed. Among the several studies, the group that underwent only splenectomy was considered the placebo group. Alternatively, rituximab was used to inhibit or prevent the production of B cells, and groups were included in the study, according to the doses used as the exposure group.

Inclusion criteria for the papers included in the analysis were as follows:

1. studies referring to at least 2 of the following eligible induction doses: ABOc or ABOi using placebo (group with splenectomy only) or rituximab 200 mg (group using 200 mg as induction during ABOi kidney transplantation), 200 to 500 mg (200–500 mg was used, or 500 mg was initially used and then later 200 mg group in 1 center), or 500 mg (first induction group was 500 mg); and
2. studies that reported 1 or more of the primary or secondary outcomes.(Supplement Table 1).

**Figure 2:**
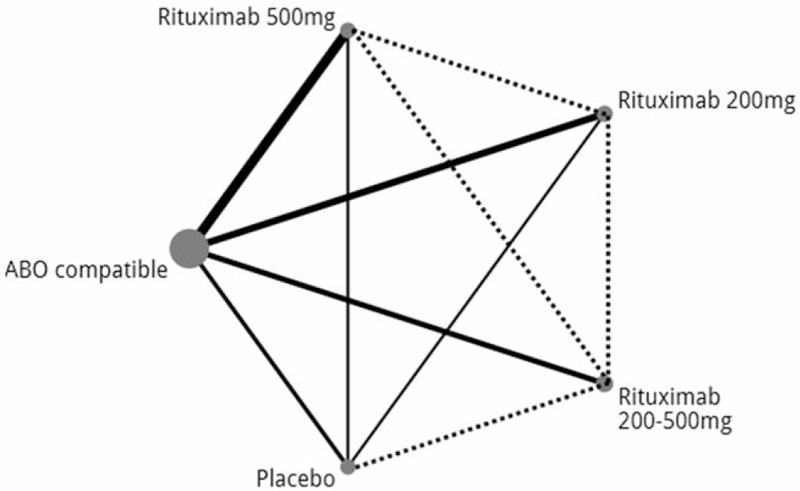
Network flow of each intervention on graft failure and patient mortality.

The primary outcome was graft survival, which was compared with ABOc KT as the reference. Secondary outcomes were survival, virus, and bacterial infection, any cause of rejection, and adverse events. (Fig. 2, network flow). This study only used published data for the eligible studies and does not require approval from an institutional review board.

### 2.4 Risk-of-bias assessment

Because randomized controlled studies were not identified in the literature search, 2 researchers (SDH and JHL) independently assessed the risk of bias of each trial using the Non-randomized Studies of Interventions (ROBINS-I) tool and the Newcastle–Ottawa Scale (NOS) for observational studies. The NOS assigns a maximum of 9 points, and studies with a total score of >7 are defined as high quality.^[15–22]^ The risk of bias, evaluated using the Newcastle–Ottawa scale, was as follows: 19.0% with 9 points for 2 studies and 9.5% with 8 points for 4 studies. Fifteen studies had a risk of bias of 71.4% with 7 points. The overall score was 7 to 9, indicating that the ROS of each study showed a good score for heterogenicity. Discrepancies were resolved by discussions between SDH and JHL (Supplement Table 3).

### 2.5 Outcome measures

We aimed to determine the effectiveness of the induction dose of rituximab against patient mortality and the occurrence of graft failure. We also investigated the potential adverse outcomes associated with induction doses and the efficacy of medications on all-cause graft rejection, biopsy-proven rejection, change in creatinine and renal function, and adverse events (such as bacterial, CMV, and BK virus infections).

### 2.6 Statistical analysis

We compared the effectiveness of 5 rituximab induction doses for ABOi KT on patient survival, graft failure, and graft rejection using Bayesian network meta-analysis. Specifically, we performed direct and indirect network meta-analysis using Bayesian models and generated rankings of different rituximab doses using generation mixed treatment comparison and Stata version 13 (StataCorp).^[23–25]^ The relative ranking probability of each treatment was estimated, and the treatment hierarchy of competing interventions was obtained using rankograms, surface under the cumulative ranking curves, and mean ranks. We performed a network meta-analysis of studies that recorded multiple treatments, which allowed estimation of the pooled effects within each treatment.^[26]^ For multiarm trials, correlations among treatment effects between arms were included in the investigations.

Studies with *j*+1 treatment arms were based on the comparison of study drug treatment effects with those of reference drugs using multivariate normal distribution, whereas treatment in usual studies were based on the homogeneity between study variances across treatments.^[27,28]^ Inconsistency tests, homogeneity analysis, and sensitivity analysis were performed using the node analysis method with the R software program. The results of inconsistency tests were assessed according to the Bayesian *P* value, where *P* < .50 was considered to indicate significant inconsistency.^[29,30]^ An *I*^2^ test was performed to assess homogeneity (*I*^2^ > 50% was considered to indicate significant heterogeneity). Furthermore, sensitivity analysis was conducted by comparing the differences between fixed-effect and random-effect models.

The clinical outcome indicators were evaluated using mean difference (MD) or odds ratio (OR) with a 95% credible interval (CrI; MD for continuous outcomes, OR for binary outcomes).^[27,31]^ When a loop connected 3 treatments, it was possible to evaluate the inconsistency between direct and indirect evidence.^[32]^ We also used the node-splitting method to calculate the inconsistency of the model, which separated evidence of a particular comparison into direct and indirect evidence.^[30]^ We then evaluated the agreement between the direct and indirect evidence and reported its Bayesian *P* value. Sensitivity analyses were conducted using the same methods after omitting data from specific studies (those with a small number of patients and events in a specific treatment arm and those with a large population that may dominate the data of specific treatment arms).^[33]^

## 3 Results

A total of 8493 records were initially retrieved from the electronic database search. After removing 1485 duplicate records, 7008 records were further excluded based on a review of either the title or abstract and 65 were retrieved for full-text review. Among these, 44 records were excluded based on the following criteria: studies involving drugs other than rituximab (n = 6); duplicated data (n = 5); patients who had not undergone KT (n = 8), hemodialysis, and peritoneal dialysis (n = 14); autosomal dominant polycystic kidney disease (n = 4); an outcome that could not be included in the statistical analysis (n = 3); review articles (n = 2); and editorial comments (n = 2).

Finally, 21 trials reporting outcomes of 4988 patients (2465 women and 2523 men) were included in the analysis (Table 1). Six studies each were conducted in the US^[34]^ and the UK,^[35]^ whereas each one was conducted in Japan (5, 28–34),^[7,36–42]^ Germany,^[43,44]^ Norway,^[45]^ Korea,^[1,14,46–50]^ and Spain.^[51]^ The number of patients per study ranged from 91 to 9180 and the median follow-up period was 2.1 (0.5–3.1) years.

**Table 1:** Relevant characteristics of included studies and proportion of patients using rituximab treatment.

References	Country/year	Rituximab treatment dose	Comparative group	Number of patients who received rituximab (n)	Mean age, years	Male patients n (%)	Prior Diabetes n (%)	Mean time on dialysis (years)
Ashimine^[29]^	Japan/2013	Rituximab 500 mg	ABOc	30	49.0 ± 15.1	17 (56.7)		
Barnett^[27]^	UK/2013	Rituximab 375 mg/m^2^	ABOc	62	46.7 ± 15.91	35 (56.5)		
Becker^[35]^	Germany/2014	Rituximab 375 mg/m^2^	ABOc	34	46 (range 18–65)	20 (59)	0 (0)	
Tanabe^[30]^	Japan/2009	Rituximab 500 mg	ABOc	24	43.0 ± 13.5	18 (75.0)	4 (16.7)	2.3 (IQR: 1.6–5.5)
Nakao^[34]^	Japan/2015	Rituximab 200 mg	Rituximab 500 mg	37	54.0 ± 3.76	20 (58.0)		
Dorje^[37]^	Norway/2015	Rituximab 375 mg/m^2^	ABOc	20	47.9 ± 12.2	15 (75.0)		
Fuchinoue^[31]^	Japan/2011	Rituximab 200 mg	Rituximab 500 mg	50	52.6 ± 12.1	34 (68)		2.6 (range 0.1–15.4)
Lee^[6]^	Korea/2015	Rituximab 200 mg	Rituximab 500 mg	95	47.68 ± 11.10	13 (68.4%)		14.38 ± 21.13
Habicht^[36]^	Germany/2011	Rituximab 375 mg/m^2^	ABOc	47	45.9 ± 1.9	33 (70.2)	2 (4.2)	2.5 ± 0.4
Moon^[42]^	Korea/2017	Rituximab 200 mg	Rituximab 500 mg	53	44.9 ± 12.0	16 (61.5)		
Hatakeyama^[28]^	Japan/2014	Rituximab 200 mg	ABOc	13	45 ± 12	8 (61.5)	1 (8)	
Hwang^[38]^	Korea/2013	Rituximab 200–500 mg	ABOc	35	44.1 ± 9.2	21 (60.0)	6 (17.1)	2.2 ± 2.9
Jeon^[39]^	Korea/2010	Rituximab 200–500 mg	ABOc	22	43.0 ± 13.2	45 (range 33–61)	10 (45.4)	
Shirakawa^[5]^	Japan/2010	Rituximab 200–500 mg	ABOc	74	43.0 ± 13.2	34 (68.2)		21.8 ± 12.4
Ko^[40]^	Korea/2016	Rituximab 200–500 mg	ABOc	248	44.2 ± 12.4	161 (64.9)	47 (19.0)	1.6 ± 3.0
Kohei^[32]^	Japan/2011	Rituximab 200 mg	ABOc	57	44.0 ± 14.8	42 (73.7)	5 (8.8)	3.7 (range: 1.3–7.2)
Kwon^[41]^	Korea/2016	Rituximab 200–500 mg	ABOc	67/167	45.7 ± 11.6	156 (66.7)	57 (24.4)	
Okumi^[33]^	Japan/2014	Rituximab 200 mg	ABOc	144	47.8 ± 13.1	91 (63.2)		2.2 (IQR: 1.2–4.8)
Park^[1]^	Korea/2015	Rituximab 200 mg	ABOc	11	49.0 ± 6.5	49.0 ± 6.5	3 (27.3)	3.2 ± 4.1
Sanchez-Escuredo^[43]^	Spain/2016	Rituximab 200 mg	ABOc	30	47 ± 134	20 (66.7)	7 (23.3)	2.3 (range: 0–13)
Schwartz^[26]^	USA/2005	Rituximab 375 mg/m^2^	ABOc	40	48.3 ± 14.8	26 (65.0)		

BMI = body mass index, eGFR = estimated glomerular filtration rate, IQR = interquartile range.

### 3.1 Effect of interventions

**Figure 3:**
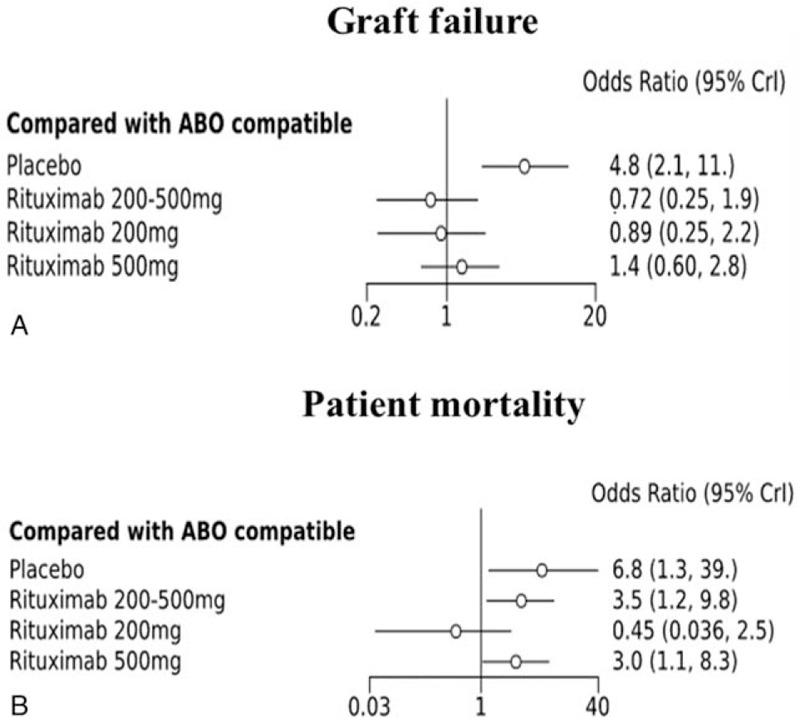
(A) Graft failure and (B) patient mortality associated with different types of induction and doses compared with ABO-compatible kidney transplantation (ABOc KT) as a reference.

The data obtained from all 21 studies (n = 4988) were included in the network analysis. The primary endpoint was graft survival. Compared with ABOc KT as the reference, the placebo group showed a significant increase in graft failure (OR, 4.8, 95% CrI: 2.1–11.0) (Fig. 3A). Other groups showed no difference in graft failure compared with ABOc KT. However, the placebo, rituximab 200 to 500 mg, and rituximab 500 mg groups were associated with higher mortality (OR, 95% CrI: 6.3, 1.3–39.0; 3.5, 1.3–9.8; and 3.0, 1.1–8.3, respectively) than the ABOc group. There was no difference in patient mortality between the ABOc KT group and the group administered a single dose of rituximab 200 mg (OR, 0.45; 95% CrI, 0.036–2.5) (Fig. 3B). We performed a sensitivity analysis in cases of graft failure, in which the included studies were subtracted one by one, and the effect on the study results was analyzed. However, the change in OR and CrI was not statistically significantly in each intervention group when ABO compatible was used as the reference (Supplement Table 2).

**Figure 4:**
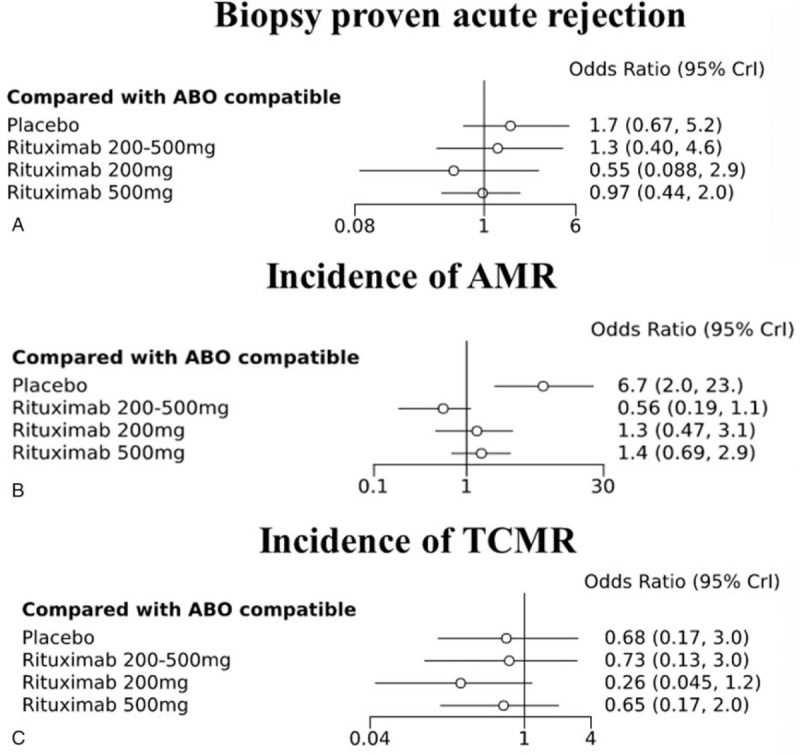
(A) Biopsy-proven acute rejection, (B) antibody-mediated rejection (AMR) and T-cell-mediated rejection (TCMR) associated with different types of induction and doses compared with ABO-compatible kidney transplantation (ABOc KT) as a reference.

We also analyzed several rejections and found that the biopsy-proven acute rejection showed no statistically significant difference in all groups compared with the ABOc KT group. Furthermore, there was no difference in glomerular filtration rate and fungal infections (Candida) compared with ABOc. Rituximab 200 mg, rituximab 200 to 500 mg, and rituximab 500 mg groups were not associated with mean difference of GFR (MD, −5.313 95% CrI: −8.36 to 2.03; −2.04, −6.34–2.49; and 2.95, −1.61–7.72, respectively). Intensive groups were not associated with fungal infections (Candida) (OR, 95% CrI: 1.6, 0.56–5.2; 0.78, 0.45–3.8; and 2.1, 0.8–4.3, respectively). However, the incidence of antibody-mediated rejection (AMR) was higher in the placebo group (OR, 6.7; 95% CrI, 2.0–23.0) and lower in the rituximab 200 to 500 mg group (OR, 0.56; 95% CrI, 0.19–1.1) than in the other groups, but the difference was not statistically significant (Fig. 4B). Furthermore, the frequency of T-cell mediated rejection (TCMR) analysis result showed no differences in incidence rates between all ABOi KT groups and the ABOc KT group, although the rituximab 200 mg group appeared to have a lower incidence rate (OR, 0.26; 95% CrI, 0.045–1.2) (Fig. 4A,C).

**Figure 5:**
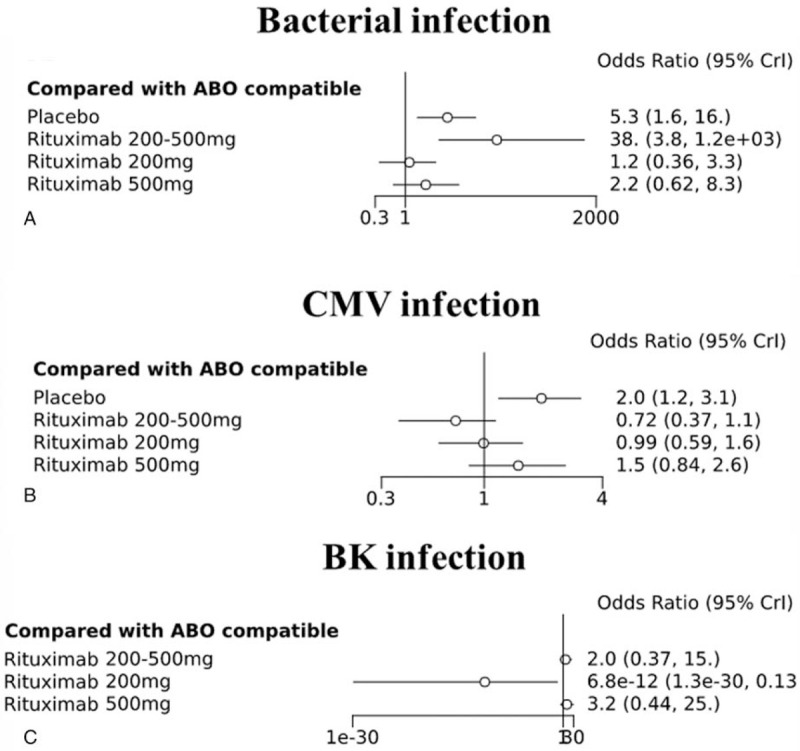
(A) Bacterial, (B) cytomegalovirus (CMV), and BK virus infections associated with different types of induction and doses compared with ABO-compatible kidney transplantation (ABOc KT) as a reference.

We analyzed the incidences of infections that could occur in relation to those infections as subgroups and the results showed that the incidence of bacterial infections was similar in the ABOc KT group and the group treated with rituximab 200 mg (OR, 1.2; 95% CrI, 0.36–3.3), but higher in the placebo and rituximab 200 to 500 mg groups (OR, 95% CrI: 5.3, 0.16–16.0, and 38.0, 3.8–1.2e+03, respectively, Fig. 5A). Similar results were also observed with the incidence of CMV infection, which was high in the placebo group but not significantly different from that in other groups (OR, 2.0; 95% CrI, 1.2–3.1). The incidence of BK infection, which is known to be closely related to graft survival, was also significantly lower in the rituximab 200 mg group than in other groups. No differences were found among the various interventions in the secondary outcome analysis, which consisted of survival, virus and bacterial infection, any cause of rejection, and adverse events (Fig. 5B,C).

**Figure 6:**
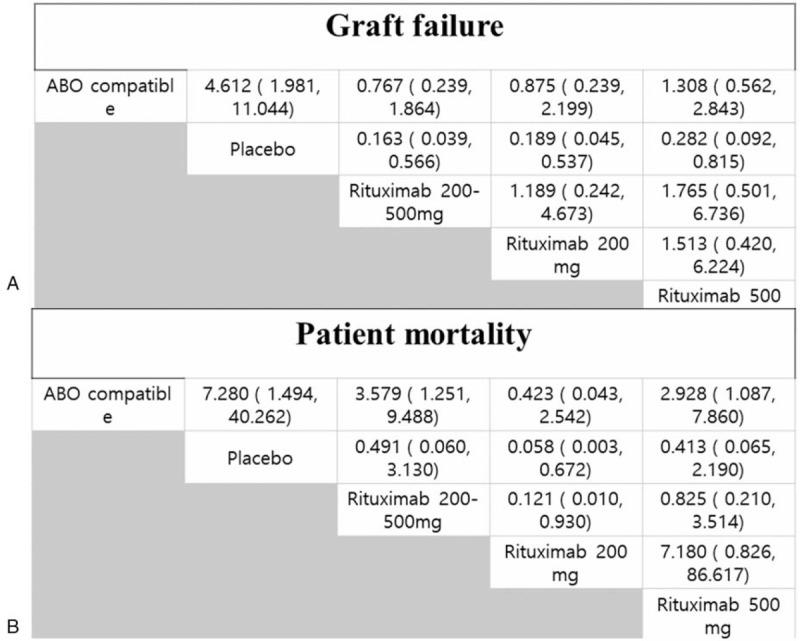
Comparison of included dose of rituximab associated with graft failure and patient mortality. Odds ratio (OR); 95% confidence interval (CI). Each cell indicates effect of column-defining intervention relative to row-defining intervention.

### 3.2 Rank probabilities

Although there was no statistically significant difference among groups, the rituximab 200 to 500 mg group ranked first and second for graft failure, with probabilities of 0.504 and 0.231, respectively. The placebo group was ranked fifth, indicating that it had the most graft failures among the intervention groups. The model-fit statistic, a deviance information criterion (DIC), was 49.6 and the residual deviance was 28.9. The rank probabilities of survival showed that the rituximab 200 mg group had the highest ranking of 0.812, followed by the ABOc KT group with, whereas the placebo group was ranked fifth with a probability of 0.734. The model-fit DIC of patient mortality was 39.6, and the residual deviance was 22.3. The network heterogeneity was estimated by comparing the common heterogeneity variance (tau [τ]) of each network with an empirical distribution of heterogeneity variances. The network of graft failure indicated substantial heterogeneity (τ = 0.65), whereas the value was τ = 0.84 for networks of patient mortality.

## 4 Discussion

In this study, we retrospectively examined the efficacy of varying induction doses of rituximab for ABOi KT. The main findings were that its effect on graft survival was not different among all groups, whereas the group treated with rituximab 200 mg showed superior mortality results than the placebo and rituximab 200 to 500 mg groups. In addition, biopsy-proven acute rejection did not differ significantly among all groups. However, the AMR showed a high incidence in the placebo group, whereas there was no statistical difference in TCMR. Infection following KT is a very important outcome for patients and rituximab 200 mg was associated with a rather low incidence of bacterial and BK infections. Moreover, the CMV infection incidence did not differ significantly among groups and there were no significant differences in all secondary outcomes.

Initially, rituximab was mostly used at a dose of 375 mg/m^2^. However, recently, 200 mg/patient or 200 to 500 mg has been frequently administered. In particular, the effect of high-dose rituximab in Asians has not been shown to differ from that in Western populations. However, because rituximab increases the frequency of infection or neutropenia, studies have investigated the use of lower doses to reduce complications, and similar effects have been observed. Some medical centers use rituximab at a dose of 100 mg/patient, and although studies have shown reasonable efficacy and manageable side effects at this dose, it has not been accepted by most transplant centers.^[36]^

The study by Nakao et al reported that the living donor KT group administered low-dose rituximab (100 mg/patient) showed no statistically significant difference in overall survival and graft survival rate compared with the group administered 200 mg/patient. The incidence of myelosuppression and viral infection with rituximab is lower at 100 mg/patient that at 200 mg/patient.^[42]^ However, various rituximab doses administered before ABOi KT have been effective in patients by maintaining grafts while reducing side effects and have also shown good cost-effectiveness. Therefore, a lower dose of rituximab is not inferior to the standard dose in efficacy or side effect-tolerability. Future studies should be conducted to confirm whether lower doses of rituximab are efficacious and safe in ABOi KT.

In this study, there was no linear relationship between rituximab dose and risk of infection. In general, a smaller dose of rituximab is expected to cause less frequent infections, but the results did not show any correlation. These results suggest that the type of immunosuppressant, duration of use, and drug concentration are different for each study. In addition, it was judged that there is a difference in opinion of whether antiviral or antibacterial agents should be administered together, and the concentration and duration are probably different for each center when administered. Although rituximab itself increases the risk of infection, we did not perform the related analysis since many factors may have a combined effect. In the future, correcting the effects of these various factors and conducting a comparative study on the dose of rituximab administered will enable accurate identification of the association of rituximab with infection. When using rituximab as an immunosuppressant for kidney transplantation, it will be possible to know which dose is associated with the best treatment outcome and the least complications.

There are some limitations to this study. First, the groups in this study were assigned according to the first rituximab dose administered. In addition, it is unclear whether the 200 to 500 mg group was similar to the 200 mg and 500 mg groups. However, this assignment was necessary because various doses were used at various transplant centers and not all doses could be specified. Second, there may have been differences in the timing of rituximab administration prior to transplantation. Some patients were administered the drug a month before KT, whereas others were treated a week before, and this difference in timing could lead to variations in the effects of rituximab. Third, treatments administered before and after rituximab may affect the frequency or severity of complications; for example, some centers administered steroids or antihistamine intravenously before rituximab and sometimes without any precautions. The frequency or severity of initial side effects may vary depending on the presence or absence of pretreatment. In addition, serious complications related to infection may vary depending on the vaccine or treatment method and frequency of local infection. Fourth, the included studies did not investigate the doses of rituximab used in KT. More randomized controlled trials will be published and should be collated and reviewed to establish criteria for the optimum dose of rituximab to be used in KT, especially ABOi KT. Finally, in the case of results related to bacterial infection, it can be seen that the range of the CrI was quite wide, which may occur due to the small sample size, and thus it may be difficult to interpret the results. However, in several cases where it is not possible to conduct randomized-controlled trial, it is difficult to present meaningful directions with a small number of patients, and therefore, meta-analysis or network meta-analysis are considered necessary.

In conclusion, the administration of low-dose rituximab shows superior efficacy to that of high-dose rituximab in ABOi KT and may exhibit better safety. Moreover, this result will facilitate the determination of the appropriate dose of rituximab for each center, which is expected to lead to more comparable outcomes between ABOi KT and ABOc KT procedures. In the future, the results of this study may be confirmed by the analysis of relevant ongoing studies once they are completed and collated.

## Author contributions

**Conceptualization:** Hee Ryong Lee, Kipyo Kim, Jin Ho Lee, Seun Deuk Hwang.

**Data curation:** Hee Ryong Lee, Kipyo Kim, Seoung Woo Lee, Joon Ho Song, Jin Ho Lee, Seun Deuk Hwang.

**Formal analysis:** Kipyo Kim, Seoung Woo Lee, Joon Ho Song, Jin Ho Lee, Seun Deuk Hwang.

**Funding acquisition:** Hee Ryong Lee, Kipyo Kim, Seoung Woo Lee, Joon Ho Song, Jin Ho Lee, Seun Deuk Hwang.

**Investigation:** Hee Ryong Lee, Kipyo Kim, Seoung Woo Lee, Joon Ho Song, Jin Ho Lee, Seun Deuk Hwang.

**Methodology:** Seoung Woo Lee, Joon Ho Song, Jin Ho Lee, Seun Deuk Hwang.

**Project administration:** Kipyo Kim.

**Resources:** Kipyo Kim, Seoung Woo Lee, Joon Ho Song, Seun Deuk Hwang.

**Software:** Kipyo Kim, Joon Ho Song, Jin Ho Lee, Seun Deuk Hwang.

**Supervision:** Kipyo Kim, Joon Ho Song, Seun Deuk Hwang.

**Validation:** Joon Ho Song, Jin Ho Lee, Seun Deuk Hwang.

**Visualization:** Kipyo Kim, Seun Deuk Hwang.

**Writing – original draft:** Hee Ryong Lee, Kipyo Kim, Seoung Woo Lee, Jin Ho Lee, Seun Deuk Hwang.

**Writing – review & editing:** Hee Ryong Lee, Seoung Woo Lee, Seun Deuk Hwang.

## Supplementary Material

Supplemental Digital Content

## Supplementary Material

Supplemental Digital Content

## Supplementary Material

Supplemental Digital Content

## Supplementary Material

Supplemental Digital Content
