# Supplementary material for: Effect of rituximab dose on induction therapy in ABO-incompatible living kidney transplantation: A network meta-analysis
Source: Medicine (Baltimore). 2021 Mar 12;100(10):e24853. doi: 10.1097/MD.0000000000024853 (PMC7969271; doi:10.1097/MD.0000000000024853)
Supplement: Supplemental Digital Content [file medi-100-e24853-s001.docx]

**Supporting Information**

**Search terms for network meta-analysis**

(((((((((((((rituximab OR rituximab)) OR ((anti AND CD20 AND monoclonal AND "abnormalities" OR "abnormalities" OR "ab"))) OR induction) OR dose) OR ("immunosuppressive agents" OR "immunosuppressive agent" OR ("immunosuppressive" AND "agents") OR "immunosuppressive agents" OR immunosuppressant)))))) AND induction) AND ((((((((((Renal Insufficiencies or Kidney Insufficiency or Insufficiency, Kidney or Kidney Insufficiencies or Kidney Failure or Failure, Kidney or Failures, Kidney or Kidney Failures or Renal Failure or Failure, Renal or Failures, Renal or Renal Failures)) OR (Chronic Renal Insufficiencies or Renal Insufficiencies, Chronic or Chronic Renal Insufficiency or Kidney Insufficiency, Chronic or Chronic Kidney Diseases or Chronic Kidney Disease or Kidney Disease, Chronic or Kidney Diseases, Chronic or Chronic Renal Diseases or Chronic Renal Disease or Disease, Chronic Renal or Diseases, Chronic Renal or Renal Disease, Chronic or Renal Diseases, Chronic)).)) OR ("renal dialysis" OR ("renal" AND "dialysis") OR "renal dialysis" OR "dialysis" OR "dialysis")) OR (("kidney transplantation"] OR ("kidney" AND "transplantation") OR "kidney transplantation")))))) AND (((("kidney transplantation" OR ("kidney" AND "transplantation") OR "kidney transplantation"))) OR (((("Arq Bras Oftalmol" OR "abo") AND **MISMATCH**])) OR ABO-incompatible kidney **TRANSPLANTATION**))))
