# Supplementary material for: Effect of rituximab dose on induction therapy in ABO-incompatible living kidney transplantation: A network meta-analysis
Source: Medicine (Baltimore). 2021 Mar 12;100(10):e24853. doi: 10.1097/MD.0000000000024853 (PMC7969271; doi:10.1097/MD.0000000000024853)
Supplement: Supplemental Digital Content [file medi-100-e24853-s002.docx]

| Comparison | Studies number | Direct evidenceOR (95% CI) | I^2^ |
| --- | --- | --- | --- |
| (ABO compatible - Placebo) | 3 | 3.84 (0.97, 14.34) | 66.4% |
| (ABO compatible - Rituximab 200-500mg) | 4 | 0.730 ( 0.242, 1.936) | 39.7% |
| (ABO compatible - Rituximab 200mg) | 5 | 0.645 ( 0.064, 2.725) | 92.6% |
| (ABO compatible - Rituximab 500mg) | 7 | 1.285 ( 0.571, 2.722) | 35.7% |
| (Placebo - Rituximab 200mg) | 2 | 0.047 ( 0.001, 0.760) | 22.5% |
| (Placebo - Rituximab 500mg) | 2 | 0.082 ( 0.002, 1.432) | 23.5% |

**Supplement Table 1: Summary information from direct comparisons of dose of ritusimab as indcuction therapy in kidney transplantation**
