# Supplementary material for: Effect of rituximab dose on induction therapy in ABO-incompatible living kidney transplantation: A network meta-analysis
Source: Medicine (Baltimore). 2021 Mar 12;100(10):e24853. doi: 10.1097/MD.0000000000024853 (PMC7969271; doi:10.1097/MD.0000000000024853)
Supplement: Supplemental Digital Content [file medi-100-e24853-s003.docx]

****Supplement Table 3****. Assessment of the risk of bias in each study using the Newcastle–Ottawa scale (NOS)

|  |  | Selection (0-4) | | | | Comparability (0-2) | | Outcome (0-3) | | |  |
| --- | --- | --- | --- | --- | --- | --- | --- | --- | --- | --- | --- |
| Type of trials | Trials | REC | SNEC | AE | DO | SC | AF | AO | FU | AFU | Total |
| Cohort studies (NOS) | Ashimine | ★ | ★ | ★ | ★ |  |  | ★ | ★ | ★ | 7 |
|  | Barnett | ★ | ★ | ★ | ★ |  |  | ★ | ★ | ★ | 7 |
|  | Becker | ★ | ★ | ★ | ★ |  |  | ★ | ★ | ★ | 7 |
|  | Tanabe | ★ | ★ | ★ | ★ | ★ |  | ★ | ★ | ★ | 8 |
|  | Nakao | ★ | ★ | ★ | ★ | ★ |  | ★ | ★ | ★ | 8 |
|  | Dorje | ★ | ★ | ★ | ★ |  |  | ★ | ★ | ★ | 7 |
|  | Fuchinoue | ★ | ★ | ★ | ★ |  |  | ★ | ★ | ★ | 7 |
|  | Lee | ★ | ★ | ★ | ★ |  |  | ★ | ★ | ★ | 7 |
|  | Habicht | ★ | ★ | ★ | ★ |  |  | ★ | ★ | ★ | 7 |
|  | Moon | ★ | ★ | ★ | ★ |  |  | ★ | ★ | ★ | 7 |
|  | Hatakeyama | ★ | ★ | ★ | ★ |  |  | ★ | ★ | ★ | 7 |
|  | Hwang | ★ | ★ | ★ | ★ |  |  | ★ | ★ | ★ | 7 |
|  | Jeon | ★ | ★ | ★ | ★ |  |  | ★ | ★ | ★ | 7 |
|  | Shirakawa | ★ | ★ | ★ | ★ |  |  | ★ | ★ | ★ | 7 |
|  | Ko ( | ★ | ★ | ★ | ★ |  |  | ★ | ★ | ★ | 7 |
|  | Kohei | ★ | ★ | ★ | ★ | ★ | ★ | ★ | ★ | ★ | 9 |
|  | Kwon | ★ | ★ | ★ | ★ | ★ |  | ★ | ★ | ★ | 8 |
|  | Okumi | ★ | ★ | ★ | ★ | ★ |  | ★ | ★ | ★ | 8 |
|  | Park | ★ | ★ | ★ | ★ |  |  | ★ | ★ | ★ | 7 |
|  | Sanchez-Escuredo | ★ | ★ | ★ | ★ |  |  | ★ | ★ | ★ | 7 |
|  | Schwartz | ★ | ★ | ★ | ★ | ★ | ★ | ★ | ★ | ★ | 9 |
| REC, representativeness of the exposed cohort; SNEC, selection of the non-exposed cohort; AE, ascertainment of exposure; DO, demonstration that the outcome of interest was not present at the start of the study; SC, study controls for age, sex, and marital status; AF, study controls for any additional factors; AO, assessment of outcome; FU, was the follow-up sufficient for outcomes to occur; AFU, adequacy of the follow-up of cohorts; | | | | | | | | | | | |
| Star means that the study was satisfied the item, and no star means the opposite situation. | | | | | | | | | | | |
