# Supplementary material for: Effect of rituximab dose on induction therapy in ABO-incompatible living kidney transplantation: A network meta-analysis
Source: Medicine (Baltimore). 2021 Mar 12;100(10):e24853. doi: 10.1097/MD.0000000000024853 (PMC7969271; doi:10.1097/MD.0000000000024853)
Supplement: Supplemental Digital Content [file medi-100-e24853-s004.docx]

| **Supplement Table 2.** Residual deviance per arm per study | | | |
| --- | --- | --- | --- |
| **Study** | **Arm** | **Residual deviance** | **Leverage** |
| 1 | ABO compatible | 1.118 | 0.893 |
|  | Placebo | 0.754 | 0.746 |
|  | Rituximab 200mg | 2.022 | 0.007 |
| 2 | Placebo | 0.901 | 0.900 |
|  | Rituximab 200mg | 0.467 | 0.463 |
| 3 | ABO compatible | 1.294 | 1.189 |
|  | Rituximab 200mg | 0.588 | 0.009 |
| 4 | ABO compatible | 1.02 | 0.873 |
|  | Rituximab 200mg | 1.364 | 1.072 |
| 5 | ABO compatible | 0.036 | 0.001 |
|  | Rituximab 200mg | 0.017 | 0.000 |
| 6 | ABO compatible | 0.037 | 0.000 |
|  | Rituximab 200mg | 0.008 | 0.000 |
| 7 | ABO compatible | 0.900 | 0.899 |
|  | Rituximab 200-500mg | 0.377 | 0.376 |
| 8 | ABO compatible | 0.035 | 0.000 |
|  | Rituximab 200-500mg | 0.010 | 0.000 |
| 9 | ABO compatible | 0.929 | 0.919 |
|  | Rituximab 200-500mg | 0.732 | 0.664 |
| 10 | ABO compatible | 0.995 | 0.978 |
|  | Rituximab 200-500mg | 0.659 | 0.597 |
| 11 | ABO compatible | 0.712 | 0.688 |
|  | Placebo | 1.170 | 0.870 |
|  | Rituximab 500mg | 1.976 | 0.010 |
| 12 | ABO compatible | 0.772 | 0.771 |
|  | Rituximab 500mg | 0.523 | 0.522 |
| 13 | ABO compatible | 0.767 | 0.766 |
|  | Rituximab 500mg | 0.640 | 0.640 |
| 14 | ABO compatible | 1.485 | 0.765 |
|  | Placebo | 1.001 | 0.645 |
|  | Rituximab 500mg | 0.332 | 0.332 |
| 16 | ABO compatible | 0.757 | 0.641 |
|  | Rituximab 500mg | 0.891 | 0.712 |
| 17 | ABO compatible | 1.126 | 0.850 |
|  | Rituximab 500mg | 0.748 | 0.432 |
| 18 | ABO compatible | 0.844 | 0.588 |
|  | Rituximab 500mg | 1.067 | 0.820 |
